# Field-Adapted Full Genome Sequencing of Peste-Des-Petits-Ruminants Virus Using Nanopore Sequencing

**DOI:** 10.3389/fvets.2020.542724

**Published:** 2020-10-26

**Authors:** Emeli Torsson, Tebogo Kgotlele, Gerald Misinzo, Jonas Johansson Wensman, Mikael Berg, Oskar Karlsson Lindsjö

**Affiliations:** ^1^Department of Biomedical Sciences & Veterinary Public Health, Swedish University of Agricultural Sciences, Uppsala, Sweden; ^2^Department of Veterinary Microbiology and Parasitology, Sokoine University of Agriculture, Morogoro, Tanzania; ^3^Department of Clinical Sciences, Swedish University of Agricultural Sciences, Uppsala, Sweden; ^4^Department of Animal Breeding and Genetics, Swedish University of Agricultural Sciences, Uppsala, Sweden

**Keywords:** peste-des-petits-ruminants virus, eradication, molecular epidemiology, full genome sequencing, MinION, miniPCR

## Abstract

Peste-des-petits-ruminants virus (PPRV) is currently the focus of a control and eradication program. Full genome sequencing has the opportunity to become a powerful tool in the eradication program by improving molecular epidemiology and the study of viral evolution. PPRV is prevalent in many resource-constrained areas, with long distances to laboratory facilities, which can lack the correct equipment for high-throughput sequencing. Here we present a protocol for near full or full genome sequencing of PPRV. The use of a portable miniPCR and MinION brings the laboratory to the field and in addition makes the production of a full genome possible within 24 h of sampling. The protocol has been successfully used on virus isolates from cell cultures and field isolates from tissue samples of naturally infected goats.

## Introduction

With the development of new and portable sequencing equipment, it is now possible to perform—in very basic laboratories—sequencing that was previously limited to well-equipped laboratories (1–4). With a small thermocycler such as the miniPCR (Amplyus, Cambridge, United States), the hand-held MinION sequencer (Oxford Nanopore Technologies, Oxford, United Kingdom), and portable computational resources, full genome sequencing and advanced molecular epidemiology can be performed in almost any setting (1–4). This is highly advantageous for the diagnosis and control of viral diseases. This approach enables rapid sequencing-based technologies in resource constrained environments, in addition to bringing the laboratory analysis closer to the disease outbreak and reducing the time from diagnosis to full genome and epidemiological investigations.

Peste des petits ruminants (PPR) is a highly contagious and deadly disease in small ruminants (5). The cause is the peste-des-petits-ruminants virus (PPRV), a single-stranded negative-sense RNA virus belonging to the genus *Morbillivirus* (6). Other morbilliviruses include canine distemper virus, measles virus, feline morbillivirus, marine morbilliviruses, and the now eradicated rinderpest virus (RPV) (7). PPR has a large socioeconomic impact, as small ruminants are mainly kept by poor and rural populations that depend on their animals for income and livelihood. Due to this, the Food and Agriculture Organization of the United Nations (FAO) and the World Animal Health Organization (OIE) have launched a control and eradication program for PPRV to eliminate the disease by 2030 (8). To reach this goal, accurate and well-functioning diagnostic and epidemiological tools need to be in place (9). The Global Strategy for Control and Eradication of PPR (8) highlights that countries in stage 2 in the eradication program (out of four stages), have to strengthen laboratory capacity with molecular methods able to better characterize the collected virus isolates (8). Use of the full genome to characterize isolates, rather than only a partial sequence or genetic marker, ensures detection of important changes within the genome (10).

PPRV is widely distributed in Africa and Asia. In many of these areas, efficient transport of samples, with an unbroken cold chain to a laboratory with the correct equipment, is hard to achieve (9, 11). A broken cold chain during sample transport risks degradation of the sensitive nucleic acid of single-stranded RNA viruses such as PPRV. Analyses performed as close to possible to the sample collection site avoids these long transports (12). More accessible, less expensive, and more timely full genome sequencing will lead to better comprehensive surveillance and detection in the control of a disease such as PPR. The implementation of these mobile methodologies for molecular epidemiology will also increase the chances for successful eradication.

Here we have developed a protocol for a quick, on-site, field-adapted full genome sequencing of veterinary significant virus diseases, with PPRV as an important example. The protocol uses the highly portable miniPCR thermocycler and the MinION sequencer.

## Materials and Methods

The full wet lab protocol is available at DOI:dx.doi.org/10.17504/protocols.io.pnxdmfn.

### Samples

A selection of samples of different origins was used to verify the protocol. These included: (i) viral RNA collected from a cell-culture grown virus (Vero-SLAM cell line), isolate Nigeria 75/1, kindly provided by Dr. Siamak Zohari, National Veterinary Institute (SVA), Uppsala, Sweden; (ii) RNA from field samples representing all currently known lineages of PPRV (cultured on the CV-1-SLAM cell line), kindly provided by Dr. William G. Dundon, International Atomic Energy Agency (IAEA), Vienna, Austria, [KP789375 (13), KR781450, KR781449 (14) and KM463083 (15)]; and, (iii) two field isolates (tissue) collected by Tebogo Kgotlele and Prof. Gerald Misinzo from an outbreak in goats in Dakawa, Morogoro region, Tanzania, in 2013 (16).

### Primer Design

Two sets of multiplex full-genome primers were designed using Primal Scheme (http://primal.zibraproject.org) (17). One primer set had an amplicon length of 800 base pairs (bp) and an overlap of 100; the other primer set had an amplicon length of 600 bp and an overlap of 40. Primers were designed using eight full genome sequences representing all known lineages available at the NCBI GenBank (Table 1). Primers, for the 600-bp and 800-bp amplicons, are available in the Supplementary Material (Tables S1, S2).

**Table 1:** Complete genomes used to generate the multiplex primers with the primal scheme.

**Accession no**.	**Lineage**	**Country**	**Year**
EU267273.1	I	Cote d'Ivoire	1989
KR781451.1	II	Cote d'Ivoire	2009
KR828814.1	II	Nigeria	2012
X74443.2	II	Nigeria	1975
KJ867540.1	III	Ethiopia	1994
KJ867543.1^*^	III	Uganda	2012
KJ867541.1	IV	Ethiopia	2010
KR828813.1	IV	Nigeria	2013

* *First genome in file*.

### RNA Extraction, cDNA Synthesis, and PCR Amplification

QIAamp Viral RNA Mini kit (Qiagen) was used according to the manufacturer's instructions to extract RNA from tissue samples from Tanzania (sample type iii). The other samples were shared with us as extracted RNA. cDNA synthesis was performed using Superscript IV First-Strand Synthesis System (Invitrogen) with 11 μl of RNA, according to the manufacturer's instructions. PCR amplification was performed using the Q5 Hot Start High Fidelity Polymerase (New England BioLabs) according to the protocol in (17). The protocol divided the multiplex primers into two pools with an even amount of primer pairs, and was run on the miniPCR thermocycler. The amplicons were then purified using AMPure XP magnetic beads (Beckman Coulter) or HighPrep PCR Clean-up System (MagBio Genomics Inc.) with a 1.8 × bead ratio and quantified using Qubit 1.0 Fluorometer dsDNA HS assay (Thermo Fisher Scientific). To verify the amplification, a 1% agarose gel electrophoresis (6–7 V/cm, 50–60 min) was performed, this is however optional in the final protocol.

### Nanopore Library Preparation and Sequencing

Sequencing libraries were prepared using the SQK-LSK109 Ligation Sequencing Kit and EXP-NBD104 Native Barcode expansion (Oxford Nanopore Technologies) according to manual and previously suggested modifications (17, 18). The purified PCR amplicons were repaired and A-tailed using the NEBNext Ultra II End Repair/dA-Tailing module (New England BioLabs). Native barcodes and adaptors were ligated to amplicons using Blunt/TA Ligase Master Mix (New England BioLabs). The library was then sequenced on a MinION Flowcell R9.4. for 10 h.

### Data Analysis

The docker, as well as guidance for replication of the study is available at (www.github.com/Ackia/Field_Seq). In addition to this, a suggested user protocol is included in the protocol at protocols.io (DOI: dx.doi.org/10.17504/protocols.io.pnxdmfn). The process in short; raw reads were basecalled using GUPPY (version 3.1.5. used for the publication. FASTQ files are available in repository PRJEB35549). Read-set composition and quality were assessed using plots produced by PycoQC (19). Demultiplexed read-sets were checked for purity using Kraken 2, and results were visualized in Pavian (20, 21). The read-sets were aligned to the reference genome (RefSeq assembly accession: GCF_000866445.1) using minimap2 (22). The resulting alignment file was sorted and converted into an index bam-file for further processing with samtools (23). BED files were created, representing the coverage of the sequence reads against the reference genome. BED files were further visualized using R and ggplot (24, 25). Consensus sequence were extracted using samtools and bcftools (23). Whole-genome comparison of sequence identity was performed using sourmash with the sequences of good quality (coverage x50 > 80%) reported from MinION sequencing (26). Based on the sourmash results, representative sequences were selected and whole genome comparison was performed between the consensus sequences produced with the FieldSeq protocol and the reference sequences using Mashtree (27). The tree from Mashtree was visualized using R and ggtree.

## Results

**Figure 1:**
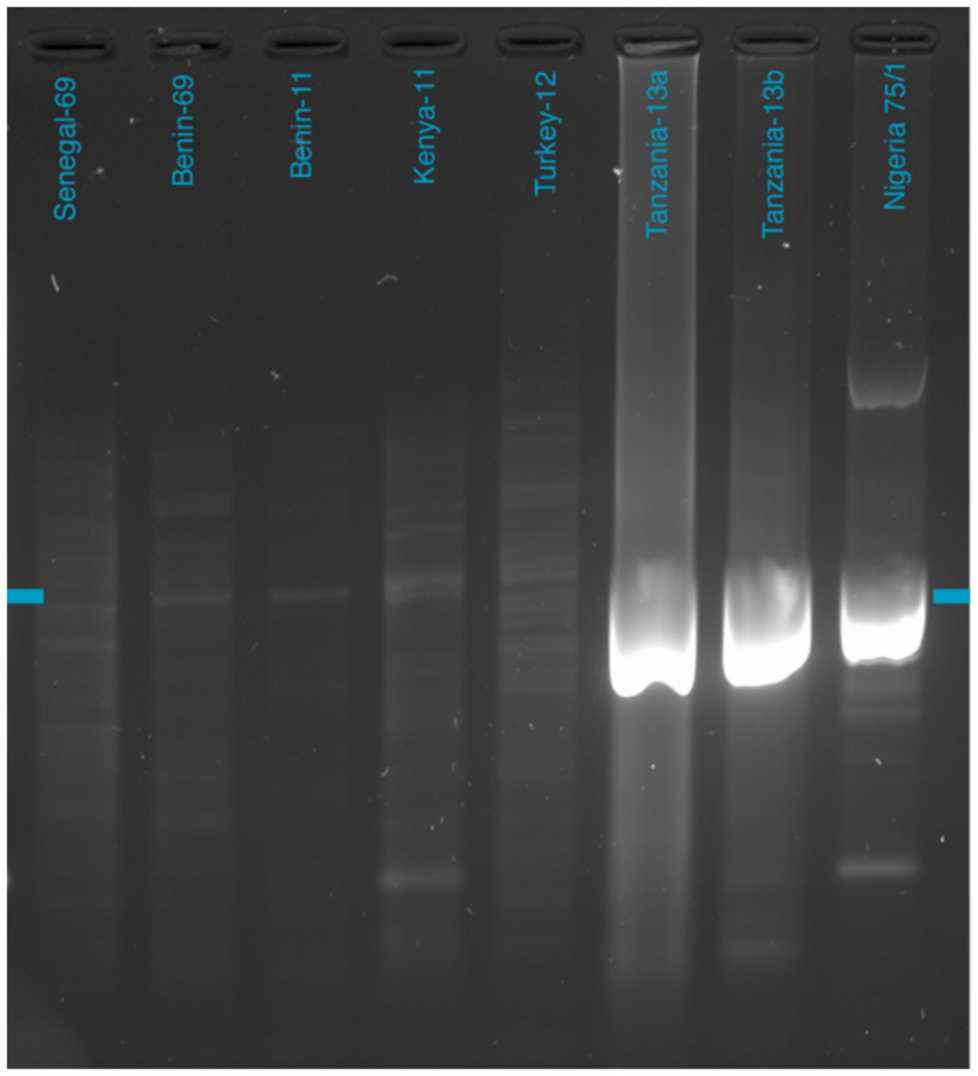
Gel electrophoresis of purified 800-bp PCR amplicons. The blue marker indicates the 800-bp size marker. Full gel image available in Supplementary Material.

Gel electrophoresis following PCR amplification of Nigeria 75/1 virus cultured on Vero-SLAM cells showed two bands—one very clear at 800 bp, and a second, weaker band at approximately 2400 bp (Figure 1). These longer amplicons are not seen on the gel electrophoresis image for the Tanzanian field samples. However, a strong band is seen at 800 bp. For the samples cultured on CV-1 cells, the gel electrophoresis image shows a narrow band at 800 bp, together with a wide selection of bands of all sizes.

Sequencing of the Nigeria 75/1 isolate produced 741,787 raw reads for the 800-bp primer set and 629,875 raw reads for the 600-bp primer set. The 800-bp primers gave a genome coverage (>50×) of 98.6% and an average coverage of 4,602 reads, whereas the 600-bp primers produced a genome coverage of 99.5%, with an average coverage of 4,586 reads (Table 2). Following this first evaluation of the primer sets, we found that the 800-bp primer set gave more even coverage of the PPRV genome, including a higher coverage of the ends of the genome. A possible explanation of this could be the increase overlap of the amplicons for the 800 bp primer set, around 100 bp instead of around 40 bp. On the basis of this result, we decided to continue working with only the 800-bp amplicon primer set for further samples (coverage comparison of both primer sets is available in Supplementary Material, Figure 1).

**Table 2:** Results from sequencing using the Oxford Nanopore MinION sequencer.

**Sample (lineage)**	**Raw reads**	**Total bp**	**N50 length (bp)**	**Reads mapped to PPRV**	**Average coverage**	**Genome coverage >50× (%)**	**Genome coverage >25× (%)**	**Source**
Nigeria 75/1^*^, 800 bp (II)	741,787	660,217,802	870	672,805	4601	98.6	99.4	Cultured on Vero-SLAM
Nigeria 75/1, 600 bp (II)	629,875	500,972,391	630	597,110	4586	99.5	99.5	Cultured on Vero-SLAM
Senegal-69 (I)	721,283	483,015,988	753	10,196	416	49.6	71.8	Cultured on CV-1^**^
Benin-69 (II)	945,266	619,883,689	826	35,716	554	78.9	87.5	Cultured on CV-1^**^
Benin-11 (II)	354,531	221,621,251	779	47,828	460	66.4	79.2	Cultured on CV-1^**^
Kenya-11 (III)	1,123,782	662,242,080	736	178,526	2311	85.0	88.8	Cultured on CV-1^**^
Turkey-12 (IV)	776,693	500,690,835	748	11,554	493	67	79.8	Cultured on CV-1^**^
Tanzania-13a (III)	947,742	707,688,820	782	771,053	4340	91.2	93.0	Field isolate
Tanzania-13b (III)	1,418,713	1,089,046,940	780	1,197,778	4506	93.5	93.5	Field isolate

* *Mean from duplicate runs*.

** *Stably transfected with a plasmid expressing the goat SLAM receptor*.

**Figure 2:**
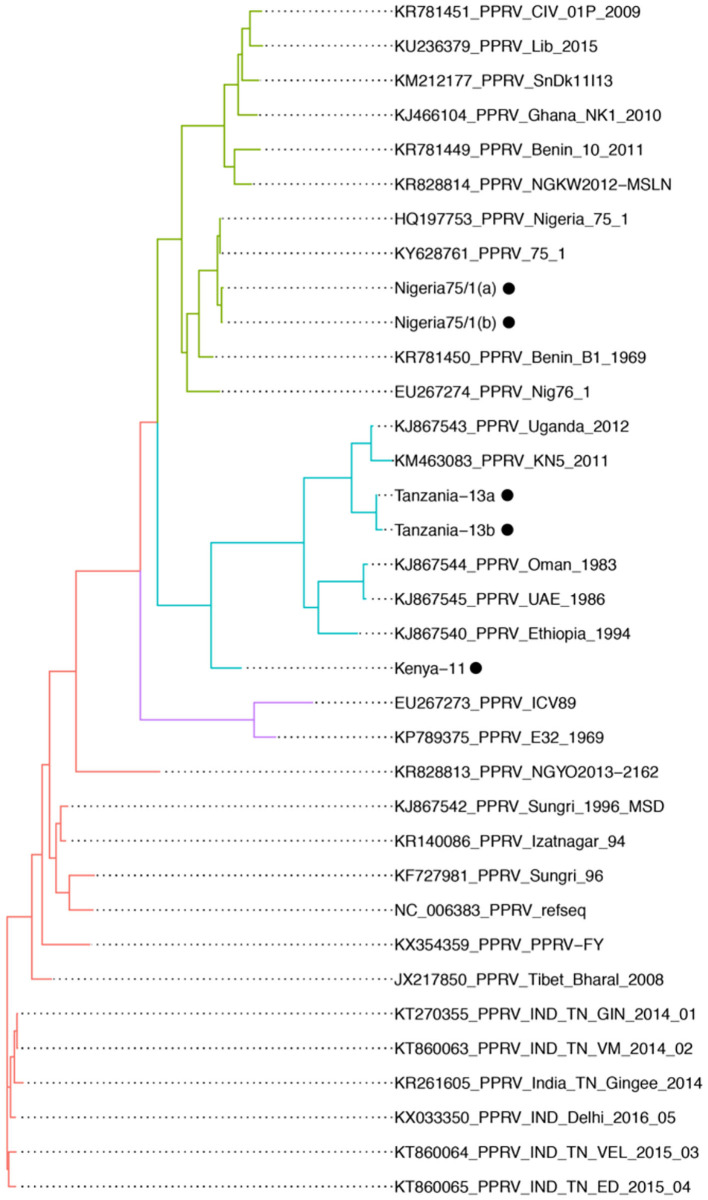
Genomic comparison of whole genome sequences of PPRV from the NCBI GenBank and the isolates with consensus sequences from the minION sequencing that produced quality sequences (>80% of the full genome). All isolates placed in the comparison according to their previously known lineage. Included consensus sequences are indicated by black dots. Isolates with purple branches indicated lineage I, isolates with green branches indicate lineage II, isolates with blue branches indicate lineage III, and isolates with red indicate lineage IV.

The Nigeria 75/1 isolate, the first trial sample, was run in duplicate to evaluate the reproducibility within a single run. The duplicates produced 709,440 and 636,171 reads that mapped against PPRV, with an average coverage of 4,454 and 4,749 reads. This was considered as an equal performance of the duplicates, which were henceforth presented as a mean of the two (Table 2). A total of 672,805 reads was mapped to the PPRV genome to give a coverage (above 50×) of 98.4% of the full genome (Table 2). For the isolates cultured on CV-1 cells, the protocol was run using the 800-bp multiplex primers. The total number of raw reads varied between 354,531 and 1,123,782; however, most reads did not map against the PPRV reference genome (Table 2). Despite this, an average of 69.4% of the genome was covered above 50×. For the two field isolates from Tanzania, the sequencing results were 947,742 and 1,418,713 raw reads, respectively, out of which 771,053 and 1,197,778 reads mapped to the PPRV reference genome (Table 2). For these isolates, 91.9% and 93.5% of the genome had coverage above x50. The whole genome sequences with good quality were compared based on nucleic acid similarity and grouped based on distance using mashtree (Figure 2). The sequences produced on MinION showed good conformity with previously sequenced genomes based on lineage and previous sequencing.

## Discussion

Here we have presented a protocol for full genome sequencing of the peste-des-petits-ruminants virus (PPRV) using the miniPCR thermocycler and Oxford Nanopore MinION. Both are suitable for use in a minimally equipped laboratory facility or even directly in the field. PPRV is currently the target of a control and eradication program, launched by the FAO and OIE in 2015, with a goal of eradication by 2030 (8). The success of this program depends on vaccination campaigns and the ability to quickly diagnose and trace the source of an outbreak (8). PPRV most often occurs in areas that lack infrastructure and laboratory facilities (11), making it difficult to reach a quick diagnosis or do adequate epidemiological investigations. Moreover, long transports of samples increase the risk of degrading the sensitive viral nucleic acid in the sample, leading to false negative results (5). By bringing the laboratory closer to the outbreak, these risks are minimized and the time from recognizing clinical signs to a molecular epidemiological investigation is significantly reduced.

**Figure 3:**
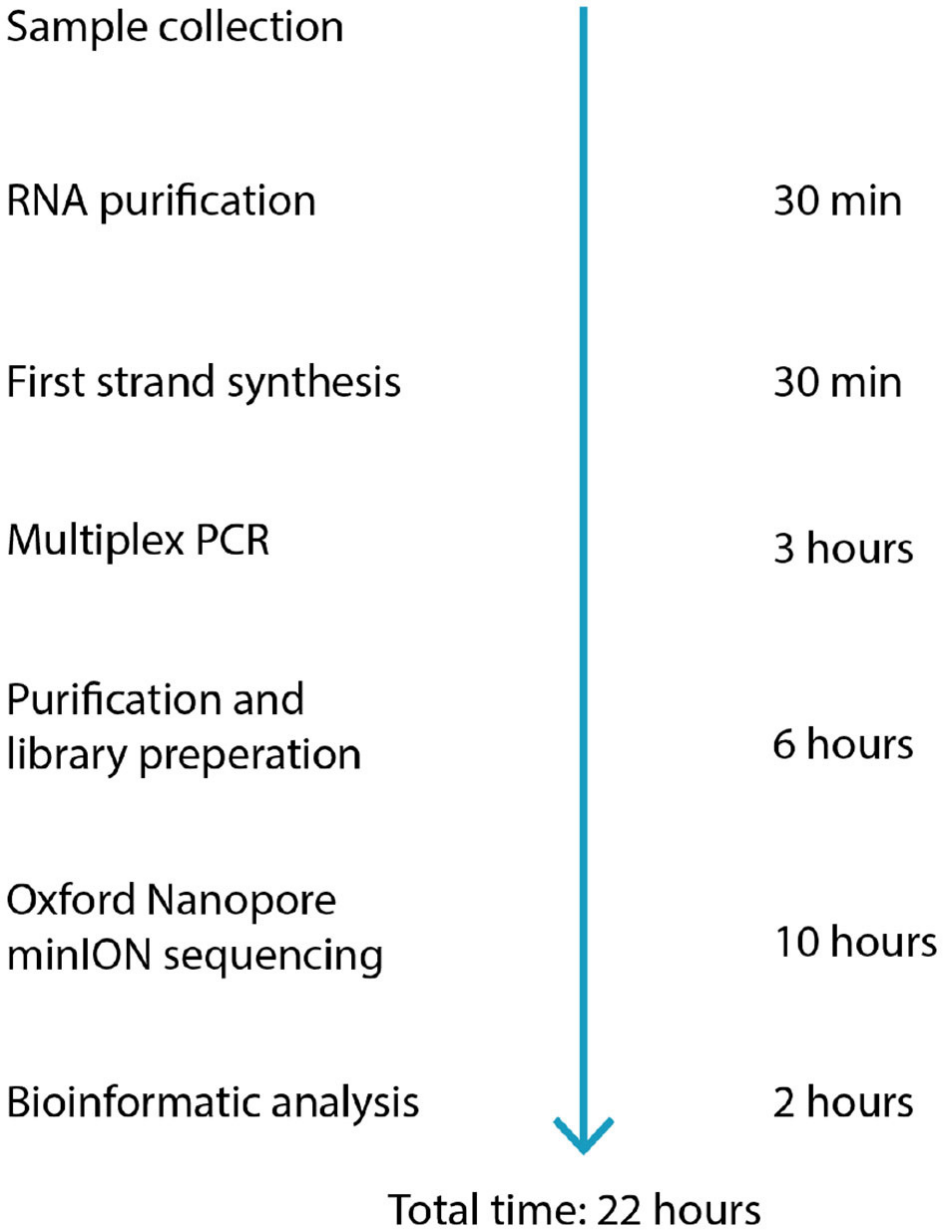
Workflow and estimated time required for each step of the protocol.

The proposed protocol does not require an expert laboratory- or sequencing technician, but it does need a basic understanding of contamination avoidance and handling of laboratory equipment. We estimate that, assuming previous training in basic pipetting skills, this protocol can easily be performed following one full run-through auscultation. The loading of reagents to the MinION flow cell requires the most practice, which can be done on used flow cells, or this single step can be performed by more experienced personnel. The time needed to run the full protocol, from the purification of RNA to analyzed sequences, is around 22–24 h (Figure 3). The protocol does not include instructions for RNA purification. In a field setting, either a spin column protocol using a small battery-driven centrifuge would be a good option or a magnetic bead-based system (as the latter is also needed in other steps of the protocol). Table 3 gives a full list of reagents and cost calculation. With our protocol, a full genome is possible to produce for under USD 100 per sample. Washing and reusing the flow cells reduces the cost even further, to around USD 80 per sample.

**Table 3:** Reagents used within the protocol, with cost calculations based on prices stated on suppliers' homepages in September 2019.

**Reagent**	**Product number**	**Source**	**Cost/unit**	**Cost/sample (USD)**
RNA extraction			variable	
SuperScript IV first-strand synthesis system	18091050	ThermoFisher Scientific	USD 2978 (200 reactions)	14.89
Multiplex primers		SigmaAldrich	variable, our 800-bp primers cost USD 158 for 100 μM/primer	0.02
Q5 hot start high-fidelity DNA polymerase	M0493L	New England Biolabs	USD 532 (500 reactions)	1.10
dNTPs (10 μM each)	R0192	ThermoFisher Scientific	USD 88 (1 ml)	0.13
HighPrep™ PCR clean-up system	AC-60050	MagBio Genomics	USD 526 (50 ml)	1.40
Qubit dsDNA HS assay kit	Q32854	ThermoFisher Scientific	USD 289 (500 reactions)	1.73
NEBNext Ultra II End Repair/dA-tailing module	E7546L	New England Biolabs	USD 795 (96 reactions)	4.10
Native barcoding expansion 1-12	EXP-PBC001	Oxford Nanopore	USD 288^*^	4
Blunt/TA ligase master mix	M0367L	New England Biolabs	USD 520 (250 reactions)	20.80
Ligation sequencing kit (incl. FlowCell priming Kit)	SQK-LSK109	Oxford Nanopore	USD 599 (6 reactions)	8.30
MinION flow cell	R9.4.1	Oxford Nanopore	USD 500–900/flow cell, depending on the quantity ordered^**^	42
Total				USD 98.5

* *Contains 12 unique barcodes and enough of each to use in 12 different sequencing libraries*.

** *Possible to wash up to 5 times, then USD 8.4/sample and total USD 81/sample (including the cost of Flow Cell Wash kit)*.

With good quality virus isolates, this protocol performed well and yielded a full genome with a mean coverage of around 4,500 reads. To standardize the quality assessment of the many new high-throughput sequences being produced, Ladner et al. suggest five standard sequenced viral genomes could be placed in (10). For molecular epidemiology, they suggest the standard “Coding complete,” which means 90–99% of the genome is sequenced with no gaps, all open reading frames (ORFs) are complete, and the average coverage is 100×. The sequences produced using our method meet these requirements when the virus isolates are of good quality.

**Figure 4:**
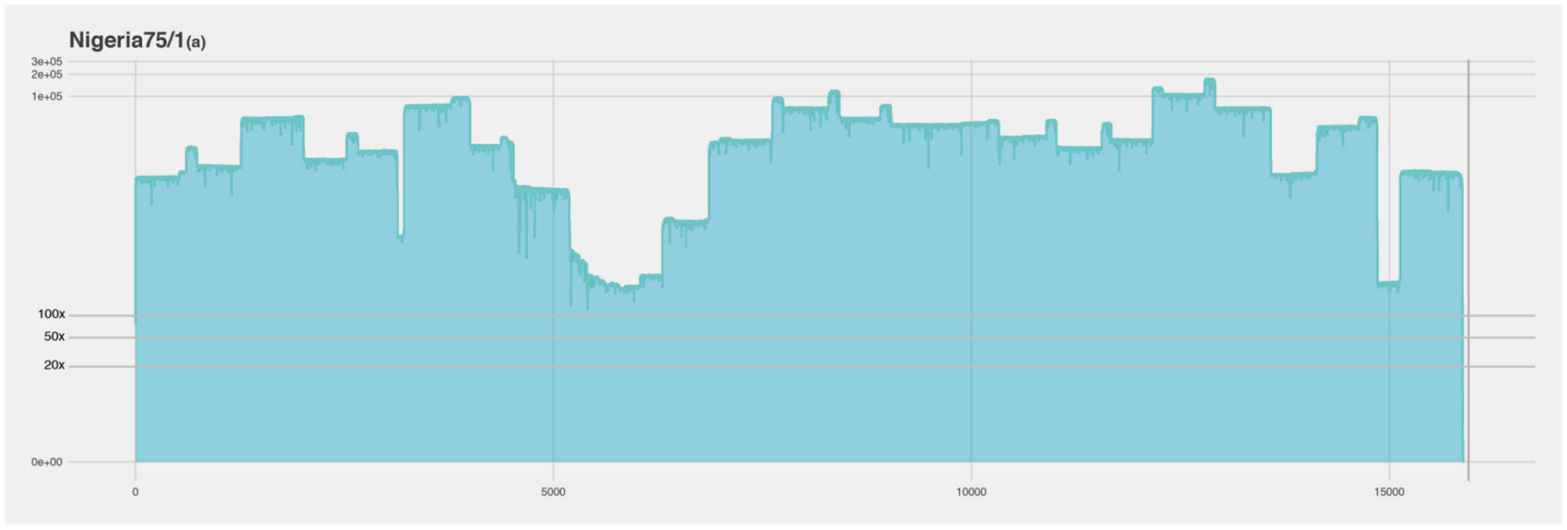
Coverage plot of Nigeria 75/1(a) duplicate. The x-axis represents the length of the genome (15.948 nucleotides). The y-axis represents the sequencing depth on a logarithmic scale. BED files, representing the coverage of the sequence reads against the reference genome, were visualized using R and ggplot.

**Figure 5:**
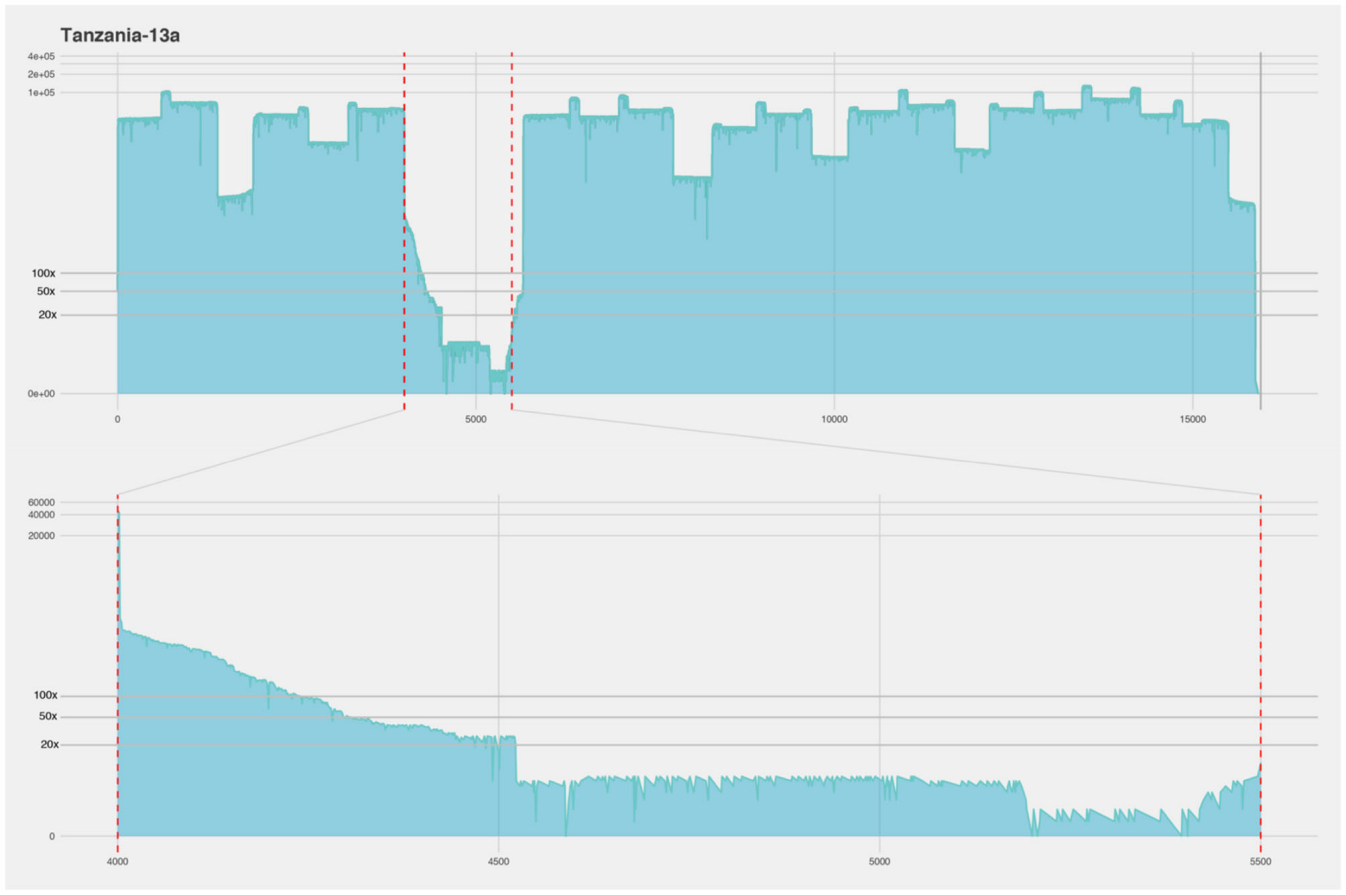
Coverage plot of the Tanzania-13a isolate. The x-axis represents the length of the genome (15.948 nucleotides). The y-axis represents the sequencing depth on a logarithmic scale. BED files, representing the coverage of the sequence reads against the reference genome, were visualized using R and ggplot. A majority of the genome was covered with over 100× sequencing depth, however in the intergenic region between the matrix and the fusion protein genes the sequencing depth falls below ×20 (framed by red dotted lines and showed in detailed in lower half of figure).

For the first run using the cell culture grown Nigeria 75/1 isolate the coverage is over 100× for the entire genome, missing only a piece of the virus poly-A tail (Figure 4). There is a slight decrease in coverage in the intergenic region between the matrix (M) and the fusion (F) protein gene (nucleotide position 4,445–5,526), as well as a short region close to the end of the genome. The M and F intergenic region is the longest intergenic region in the PPRV genome and is rich in GC content and secondary structures (28). These properties makes the region difficult for both primer design and amplification. This region have the lowest coverage in all the sequenced isolates, and was problematic for both studied primer sets. In the isolate from Tanzania it is the only region with low coverage (Figure 5), however the coverage is above zero and for molecular epidemiology the ORF are of most importance (10).

**Figure 6:**
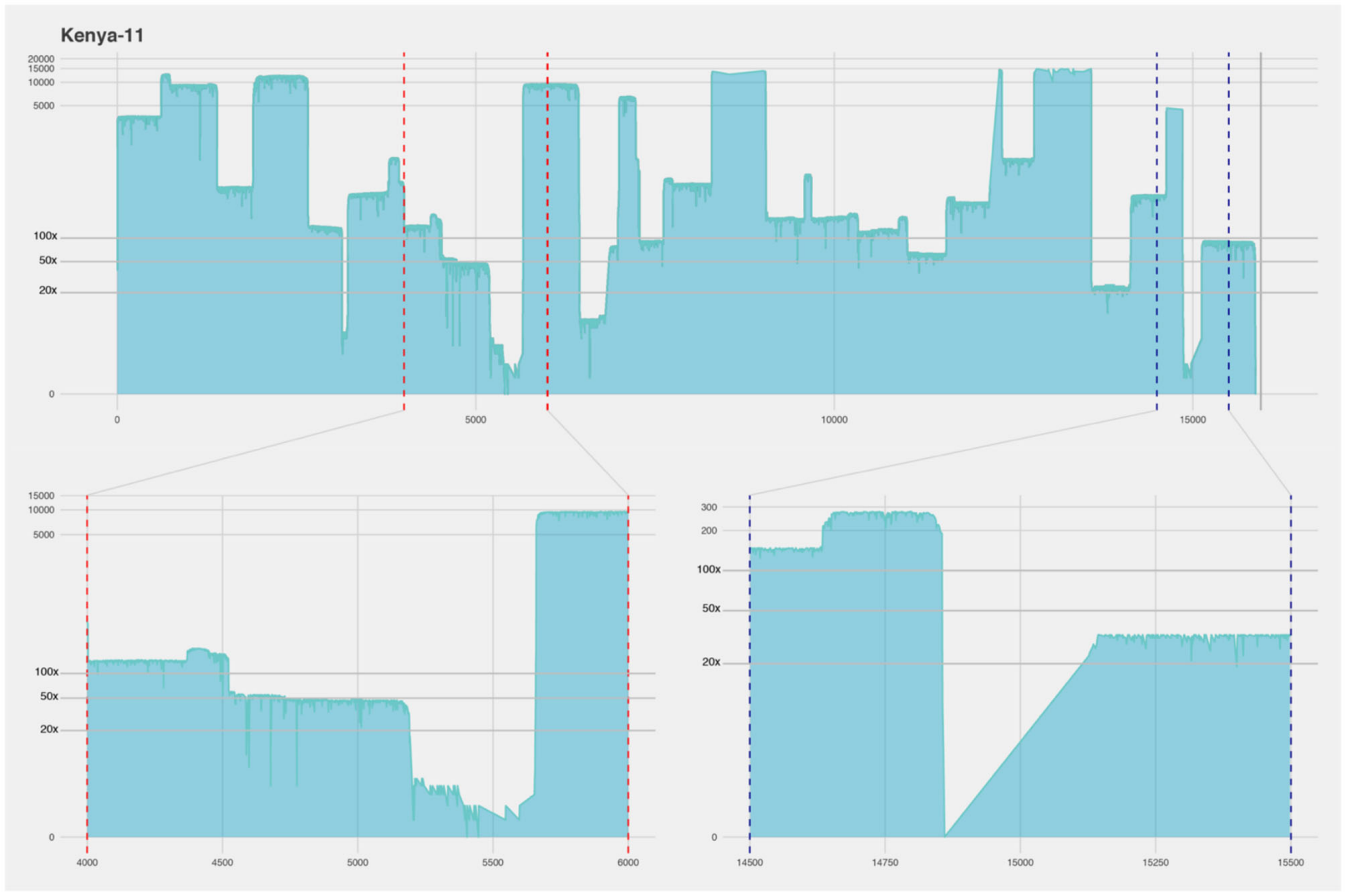
Coverage plot of the Kenya-11 isolate cultured on CV-1 cells. The x-axis represents the length of the genome (15.948 nucleotides). The y-axis represents the sequencing depth on a logarithmic scale. BED files, representing the coverage of the sequence reads against the reference genome, were visualized using R and ggplot. The coverage of this isolate was more uneven, however 85% was covered with ×50 sequencing depth. The lower part of the figure shows a detailed view of two regions with lower coverage, the intergenic region between the matrix and the fusion protein genes (framed by red dotted lines) and a region close to the end of the genome within the large protein gene (framed by blue dotted lines).

In the isolates cultured on CV-1 cells, we did not get equally good coverage over the full genome as we did for the Nigeria 75/1 and Tanzanian isolates (Figure 6, Table 2). The majority of the reads from the CV-1 samples instead mapped against the human genome. We suspect this is due to the low concentration of viral RNA, degradation of the viral genomes in the samples, and that the human sequences were mistakenly interpreted as such but in fact, had originated from the CV-1 cells (African Green monkey kidney cells). Even though this is not a perfect result, it shows how this protocol works with degraded and damaged samples. Despite the reduced coverage of the genome, we were able to extract 49.6–85.0% (with >50× coverage) of the full genomes in these five samples with an average coverage well above 100× for them (Table 2). The regions with lowest coverage for these isolates were the same for these as for the isolates of better quality, the M-F intergenic region and a region toward the end of the genome within the large protein, exemplified by the Kenya-11 isolate in Figure 6. Coverage plots for all sequenced isolates are available as Supplementary Material.

The four samples that produced above 80% of the full genome (Nigeria 75/1, Tanzania-13a/b, and Kenya-11) were used in a genomic comparison together with other available whole genomes (Figure 2). The Nigeria 75/1 isolate that performed excellent in the protocol placed together with the Nigeria 75/1 sequence collected from the database. The isolate from Kenya (Kenya-11) was previously sequenced with the accession number KM463083 (15) which is also included in the comparison. These two whole genome sequences is slightly seperated. This is probably due to the sequences produced using the protocol suggested here is not covering 100% of the genome, wheras the published sequence is full and produced by Sanger sequencing. They do, however, place within the same branch, together with other islolates from lineage III of PPRV. Within the same branch, the two samples from Tanzania (-13a and -13b) are also placed closed together, as expected due to the samples being collected from the same outbreak. By comparing, the consensus sequences produced by the described protocol with previously published sequences produced using the other sequencing techniques; we were able to evaluate the performance of the protocol. Other comparisons of the minION sequencing technique to other more traditional, and labor and equipment intensive have equally found that the method produces high quality sequences (29).

A common practice is to use only the genetic marker, the partial nucleoprotein sequence, to study the phylogeny of a PPRV isolate, as these 255 nts is what the lineage is based on. This increases the risk of missing important changes in the genome outside of the marker, but these changes could be important in the transmission routes and the virus evolution (10). Using the full genome also enables the use of advanced phylogenies such as those produced by alignments with VIRULIGN (30). The isolates used to verify our protocol are from very different timepoints and geographic regions. If the sequences had belonged to an ongoing outbreak within the same area, this improved resolution of the comparison could help determine the start and transmission route of the outbreak. It would also have made it possible to track the outbreak in real-time using tools such as Nextstrain (12, 31). For such analyses during outbreaks, the viruses need to be thoroughly sequenced. With our protocol, the production of complete genomes from PPRV field isolates are simplified and will hopefully lead to more full genomes being produced and published.

The use of full genome sequencing for epidemiology and disease surveillance is dependent on the sharing of data and the uploading of the sequences to freely available databases. A genome sequence viewed in isolation can only give limited information (1). Currently, there are 74 complete PPRV genomes available in the NCBI GenBank. Only two are isolated from a wild ruminant: a Dorcas gazelle from a zoological collection in the United Arab Emirates in 1986 (32, 33), and a Capra Ibex in China in 2015 (34). One of the questions in PPR epidemiology is the role of wild ruminants in the spread of the disease. Identified cases in African wildlife are so far considered to be spill-overs from domestic animals, but outbreaks of PPR have occurred several times in Asian wildlife (35). With additional full genome sequences available, this question could possibly be solved.

In conclusion, we have presented a field-adapted, easy to follow, protocol for full genome sequencing of PPRV using the miniPCR thermocycler and the MinION sequencer. With high-quality isolates, the protocol produces a near-complete genome for < USD 100 per sample. We hereby hope to increase the number of complete genomes available for PPRV. More genomes would allow evaluation of the virus evolution and more precise molecular epidemiological investigations. In addition, they would provide a basis for vaccine and drug development (3).

## Data Availability Statement

The datasets generated for this study can be found in the European Nucleotide Archive Database under accession number: PRJEB35549.

## Author Contributions

Conceptualization: ET and OK. Formal analysis: ET, TK, and OK. Writing—original draft preparation: ET. Writing—review and editing: OK, JJ, MB, and ET. Visualization: OK. Supervision: MB, GM, and JJ. Funding acquisition: ET, JJ, and OK. All authors contributed to the article and approved the submitted version.

## Conflict of Interest

The authors declare that the research was conducted in the absence of any commercial or financial relationships that could be construed as a potential conflict of interest.
