# Supplementary material for: Field-Adapted Full Genome Sequencing of Peste-Des-Petits-Ruminants Virus Using Nanopore Sequencing
Source: Front Vet Sci. 2020 Oct 26;7:542724. doi: 10.3389/fvets.2020.542724 (PMC7649132; doi:10.3389/fvets.2020.542724)
Supplement: Supplementary file 1 [file Data_Sheet_1.docx]

Table S1. Primer sequences for 800 base pair amplicons

| Primer | Sequence | Pool | Tm | GC% | Start | End | Amplicon size |
| --- | --- | --- | --- | --- | --- | --- | --- |
| PPRV_1_LEFT | CCAAACAAAGTTGGGTAAGGATAGATCT | 1 | 61.22 | 39.29 | 1 | 29 | 740 |
| PPRV_1_RIGHT | AAAGCGGAATTCCCCAATCACC | 1 | 61.47 | 50.00 | 740 | 718 |  |
| PPRV_2_LEFT | CCTCCATACTGGCACAAGTTTGG | 2 | 61.68 | 52.17 | 609 | 632 | 792 |
| PPRV_2_RIGHT | ATGCATGTGACTCTCCCTCTCC | 2 | 61.55 | 54.55 | 1401 | 1379 |  |
| PPRV_3_LEFT | TCACAGCAGAGGAAGCCAAACT | 1 | 62.14 | 50.00 | 1263 | 1285 | 762 |
| PPRV_3_RIGHT | GATTTCGCCTGATCCGATTGCT | 1 | 61.38 | 50.00 | 2025 | 2003 |  |
| PPRV_4_LEFT | AGAGATACCCTTGAAAGCTGGAGG | 2 | 61.67 | 50.00 | 1893 | 1917 | 770 |
| PPRV_4_RIGHT | GGCAACTCCTGGATCGTCTTTG | 2 | 61.51 | 54.55 | 2663 | 2641 |  |
| PPRV_5_LEFT | ACGGTGACAGAGTGTTCATCGA | 1 | 61.65 | 50.00 | 2526 | 2548 | 816 |
| PPRV_5_RIGHT | GGTGCATTACGGTTGCTTAGCA | 1 | 61.77 | 50.00 | 3342 | 3320 |  |
| PPRV_6_LEFT | CAAGCAAGCTCAACATTGATCACA | 2 | 60.56 | 41.67 | 3214 | 3238 | 792 |
| PPRV_6_RIGHT | ATATTGAAGGCGACGGCATTGG | 2 | 61.64 | 50.00 | 4006 | 3984 |  |
| PPRV_7_LEFT | CGCTGTCTTTGTTAACACCTTGGA | 1 | 61.74 | 45.83 | 3789 | 3813 | 734 |
| PPRV_7_RIGHT | GGGAGCCTTGGAGTGTGTTTTT | 1 | 61.28 | 50.00 | 4523 | 4501 |  |
| PPRV_8_LEFT | TCAGTACCCCAAGAATTCCGTGT | 2 | 61.58 | 47.83 | 4370 | 4393 | 822 |
| PPRV_8_RIGHT | TTTTTGTGGCGGGGTTGGATTC | 2 | 62.18 | 50.00 | 5192 | 5170 |  |
| PPRV_9_LEFT | GAAGACACCCAACCACCGAAAC | 1 | 61.89 | 54.55 | 4971 | 4993 | 816 |
| PPRV_9_RIGHT | ACAGAGCATCCTCGACAGGTTT | 1 | 61.67 | 50.00 | 5787 | 5765 |  |
| PPRV_10_LEFT | CGAGCCAACAAACCCTGGTTAT | 2 | 61.14 | 50.00 | 5658 | 5680 | 787 |
| PPRV_10_RIGHT | CATTCTTGTGCCCCGATGTTGT | 2 | 61.72 | 50.00 | 6445 | 6423 |  |
| PPRV_11_LEFT | AAAGGCCCGAGTCACCTATGTG | 1 | 62.32 | 54.55 | 6307 | 6329 | 812 |
| PPRV_11_RIGHT | TGAGCCCTGGGTTGATCTTAGG | 1 | 61.49 | 54.55 | 7119 | 7097 |  |
| PPRV_12_LEFT | CCCTTCAGCGGCAATTTGTACA | 2 | 61.71 | 50.00 | 6983 | 7005 | 770 |
| PPRV_12_RIGHT | GGAGGATTCATACACCACCGGA | 2 | 61.55 | 54.55 | 7753 | 7731 |  |
| PPRV_13_LEFT | TCATCGGTGATGAAGTCGGCAT | 1 | 62.11 | 50.00 | 7617 | 7639 | 807 |
| PPRV_13_RIGHT | CTACCCAATTGGCCTCGTTGTC | 1 | 61.51 | 54.55 | 8424 | 8402 |  |
| PPRV_14_LEFT | ACTTAATCTAGCCGGGCCTACTC | 2 | 61.32 | 52.17 | 8293 | 8316 | 752 |
| PPRV_14_RIGHT | GCCAGGGGAAACACTCTATCCT | 2 | 61.49 | 54.55 | 9045 | 9023 |  |
| PPRV_15_LEFT | ATGTTTCCAGGAGCGATCATGC | 1 | 61.33 | 50.00 | 8913 | 8935 | 774 |
| PPRV_15_RIGHT | TGCCGTTGACTCTTCTGAGACA | 1 | 61.59 | 50.00 | 9687 | 9665 |  |
| PPRV_16_LEFT | TCTGTTTTATATCAGGGACGGCTG | 2 | 60.44 | 45.83 | 9565 | 9589 | 765 |
| PPRV_16_RIGHT | TCCCCAGTTAAGTGTATATCGTGGG | 2 | 61.68 | 48.00 | 10330 | 10305 |  |
| PPRV_17_LEFT | GGTGCATTCTTGGATCACTGCT | 1 | 61.20 | 50.00 | 10190 | 10212 | 831 |
| PPRV_17_RIGHT | CGCCATTCCATTGTCTCGGAAA | 1 | 61.51 | 50.00 | 11021 | 10999 |  |
| PPRV_18_LEFT | AGAGATAAAAGAGACAGGGCGACT | 2 | 61.12 | 45.83 | 10897 | 10921 | 788 |
| PPRV_18_RIGHT | CTGCTCTAGATGCCTCCCTCTT | 2 | 61.02 | 54.55 | 11685 | 11663 |  |
| PPRV_19_LEFT | GCCCATGAGAGCGGAGTAAGAA | 1 | 62.12 | 54.55 | 11561 | 11583 | 728 |
| PPRV_19_RIGHT | GCAGAATAAGGGTCACTTGCCC | 1 | 61.52 | 54.55 | 12289 | 12267 |  |
| PPRV_20_LEFT | AGCCGACCTGAAGAGGATGATA | 2 | 60.42 | 50.00 | 12169 | 12191 | 749 |
| PPRV_20_RIGHT | GCACACGAAGAGCTGAAGTCTC | 2 | 61.49 | 54.55 | 12918 | 12896 |  |
| PPRV_21_LEFT | TGGCTATCTTATCAAACGCCATGAG | 1 | 61.26 | 44.00 | 12787 | 12812 | 797 |
| PPRV_21_RIGHT | AAAGCTGACTTGTTGACCAGGT | 1 | 60.36 | 45.45 | 13584 | 13562 |  |
| PPRV_22_LEFT | CGAATGAGCTATGTACAAACCCCTT | 2 | 61.15 | 44.00 | 13452 | 13477 | 818 |
| PPRV_22_RIGHT | AGGATCAGTGTTCCATGAGGCC | 2 | 62.08 | 54.55 | 14270 | 14248 |  |
| PPRV_23_LEFT | CCGATGTGTATTGCAGCTCCAA | 1 | 61.26 | 50.00 | 14133 | 14155 | 723 |
| PPRV_23_RIGHT | CCCAGGTTACTTCAGGTTTGCC | 1 | 61.40 | 54.55 | 14856 | 14834 |  |
| PPRV_24_LEFT | GCAGAGCCGGACAAAGAGAAAT | 2 | 61.20 | 50.00 | 14736 | 14758 | 762 |
| PPRV_24_RIGHT | TGAAATGAGCCAGCTCCCTGTA | 2 | 61.42 | 50.00 | 15498 | 15476 |  |
| PPRV_25_LEFT | AGTAATTTCATTTCAACTGAGTGTTACCTT | 1 | 60.20 | 30.00 | 15128 | 15158 | 749 |
| PPRV_25_RIGHT | TTTCATGGTGGAGGAGAAGGGG | 1 | 61.70 | 54.55 | 15877 | 15855 |  |

Table S2 Primer sequences for 600 base pair amplicons

| Primer | Sequence | Pool | Tm | %GC |
| --- | --- | --- | --- | --- |
| PPRV_1_LEFT | CCAAACAAAGTTGGGTAAGGATAGATCT | 1 | 61.21 | 39.28 |
| PPRV_1_RIGHT | CAGTTGATCCTTTTCCTGCTCCC | 1 | 61.43 | 52.17 |
| PPRV_2_LEFT | CATCACGTGGTGCTGATTTGGA | 2 | 61.45 | 50.0 |
| PPRV_2_RIGHT | GCAACTTCGCCTAGCTGTTGAT | 2 | 61.50 | 50.0 |
| PPRV_3_LEFT | TTGAAACCATGTATCCTGCGCT | 1 | 60.61 | 45.45 |
| PPRV_3_RIGHT | GGCATGATTTCCAGGAGCAGTT | 1 | 61.20 | 50.0 |
| PPRV_4_LEFT | GGCCCGACGAAAAGGACAAAAC | 2 | 62.76 | 54.54 |
| PPRV_4_RIGHT | ATTTCGCCTGATCCGATTGCTG | 2 | 61.63 | 50.0 |
| PPRV_5_LEFT | GTGCAGCACCGAACCCTGATAT | 1 | 62.95 | 54.54 |
| PPRV_5_RIGHT | AACACTCTGTCACCGTTCCAGA | 1 | 61.53 | 50.0 |
| PPRV_6_LEFT | GAAAACCTTGCGAGTCCCTGAA | 2 | 61.06 | 50.0 |
| PPRV_6_RIGHT | ACAGCAGGTTTCTTGAGGACCT | 2 | 61.55 | 50.0 |
| PPRV_7_LEFT | GACCCAACATCCGAGGTTGAGT | 1 | 62.25 | 54.54 |
| PPRV_7_RIGHT | GGGTATCAGTCGACCATCGTGA | 1 | 61.58 | 54.54 |
| PPRV_8_LEFT | TGACCGAGATCTATGACTTCGACA | 2 | 60.92 | 45.83 |
| PPRV_8_RIGHT | TTCTCCCATGAGCCGACTATGT | 2 | 60.95 | 50.0 |
| PPRV_9_LEFT | TAGAGTTCCGCTCAGCCAATGC | 1 | 62.93 | 54.54 |
| PPRV_9_RIGHT | GGGAGCCTTGGAGTGTGTTTTT | 1 | 61.27 | 50.0 |
| PPRV_10_LEFT | TCAAGGCCTGTTCAAGATCTTGT | 2 | 60.18 | 43.47 |
| PPRV_10_RIGHT | GTTTCGGTGGTTGGGTGTCTTC | 2 | 61.89 | 54.54 |
| PPRV_11_LEFT | AAACTTAGGGGCCAAGTCCACA | 1 | 61.83 | 50.0 |
| PPRV_11_RIGHT | GTTTACGCATGGATGTGCAGGA | 1 | 61.51 | 50.0 |
| PPRV_12_LEFT | AGGACCACCTTCCAACAAGACA | 2 | 61.49 | 50.0 |
| PPRV_12_RIGHT | ACTGACAGAGCATCCTCGACAG | 2 | 61.77 | 54.54 |
| PPRV_13_LEFT | ACAGCCATCGACAATTGTACGA | 1 | 60.35 | 45.45 |
| PPRV_13_RIGHT | GGGCCTTTATCCCCTTGCTTTC | 1 | 61.54 | 54.54 |
| PPRV_14_LEFT | TCAGTTATGCACTAGGTGGAGATATCA | 2 | 61.13 | 40.74 |
| PPRV_14_RIGHT | CCGACCTGTATCGTCACTCCAT | 2 | 61.58 | 54.54 |
| PPRV_15_LEFT | GGAGACGATTATCAGCCAAGATCC | 1 | 60.85 | 50.0 |
| PPRV_15_RIGHT | GCGGACATGGTGATTGACCTTT | 1 | 61.45 | 50.0 |
| PPRV_16_LEFT | CCGATCCTCAACCTGATCCCAT | 2 | 61.61 | 54.54 |
| PPRV_16_RIGHT | AATCGACTTATCCGCGGCTTTG | 2 | 61.62 | 50.0 |
| PPRV_17_LEFT | ACCTCCGGTGGTGTATGAATCC | 1 | 61.80 | 54.54 |
| PPRV_17_RIGHT | TTACAACCGCAAGAGGCTCTCT | 1 | 61.66 | 50.0 |
| PPRV_18_LEFT | GTGGGGGAATTGAAGCTGACAG | 2 | 61.46 | 54.54 |
| PPRV_18_RIGHT | AAGCCCTCTGATCTCTGTGGTC | 2 | 61.48 | 54.54 |
| PPRV_19_LEFT | CCACCTTATAAGCAGTCATTTCTGGG | 1 | 61.63 | 46.15 |
| PPRV_19_RIGHT | GGCTGAGTTGTGCTGTTGAGTG | 1 | 62.19 | 54.54 |
| PPRV_20_LEFT | AGAGCTGCGACCATCAATCAAG | 2 | 60.99 | 50.0 |
| PPRV_20_RIGHT | CCACTGAGAGCTATGCATGTGC | 2 | 61.63 | 54.54 |
| PPRV_21_LEFT | AAGAGTCAACGGCAAAATGGGC | 1 | 62.22 | 50.0 |
| PPRV_21_RIGHT | TGTCAACGCCACAATGTTCCAG | 1 | 61.89 | 50.0 |
| PPRV_22_LEFT | ACTTCAGGATGTCACTCTGGAGT | 2 | 60.75 | 47.82 |
| PPRV_22_RIGHT | TCCCACTCATTTTTCAGTGCAGC | 2 | 61.86 | 47.82 |
| PPRV_23_LEFT | TCGTTGGATTCAGGTTTGGCTG | 1 | 61.39 | 50.0 |
| PPRV_23_RIGHT | CTGGTTGGCATCGCAGATAGTT | 1 | 61.00 | 50.0 |
| PPRV_24_LEFT | CGCAAGCCAAAAGATGAGGTCA | 2 | 61.70 | 50.0 |
| PPRV_24_RIGHT | CTGCTCTAGATGCCTCCCTCTT | 2 | 61.02 | 54.54 |
| PPRV_25_LEFT | ATCGCCTCTCTAGTGCAAGGTG | 1 | 62.10 | 54.54 |
| PPRV_25_RIGHT | TGGCATTAGTCCAGCGTCTATCA | 1 | 61.50 | 47.82 |
| PPRV_26_LEFT | ACCTCAACATGAGCAGGTTATTTGT | 2 | 60.98 | 40.0 |
| PPRV_26_RIGHT | TCCTCAGGGCTATAGCAAGCTG | 2 | 61.60 | 54.54 |
| PPRV_27_LEFT | TCCGGTATAACATTGCTGACAAAGA | 1 | 60.56 | 40.0 |
| PPRV_27_RIGHT | TATTTCACCTGGGTGCTCCGAT | 1 | 61.48 | 50.0 |
| PPRV_28_LEFT | GAGCCGACATCACCCTAGATGA | 2 | 61.33 | 54.54 |
| PPRV_28_RIGHT | ATGTCGTCATCGCCAATCAAGG | 2 | 61.31 | 50.0 |
| PPRV_29_LEFT | GCTAAGTCTACAGCAATGTCCATGA | 1 | 60.91 | 44.0 |
| PPRV_29_RIGHT | TTGGAGCTGCAATACACATCGG | 1 | 61.25 | 50.0 |
| PPRV_30_LEFT | AAGTGATGAGGACGTGGTCACA | 2 | 61.60 | 50.0 |
| PPRV_30_RIGHT | CATAGAACCGGAACCTTCCCCT | 2 | 61.48 | 54.54 |
| PPRV_31_LEFT | GTTATAAAGCTGTAGAGATCTCCACGT | 1 | 60.46 | 40.74 |
| PPRV_31_RIGHT | TCGGATAGACAAGCAGGCTCTC | 1 | 61.59 | 54.54 |
| PPRV_32_LEFT | ATCAAAGTTATGCCTATTAGCGGAGA | 2 | 60.23 | 38.46 |
| PPRV_32_RIGHT | TAATTCGCGTTGCCTGCTACTG | 2 | 61.55 | 50.0 |
| PPRV_33_LEFT | ACCCCTATCGAGAAAATCCTAATTAATTGT | 1 | 60.84 | 33.33 |
| PPRV_33_RIGHT | GATTGCCAGTGGAATTTCATGGTG | 1 | 60.91 | 45.83 |
