# Supplementary figures and images for: Field-Adapted Full Genome Sequencing of Peste-Des-Petits-Ruminants Virus Using Nanopore Sequencing
Source: Front Vet Sci. 2020 Oct 26;7:542724. doi: 10.3389/fvets.2020.542724 (PMC7649132; doi:10.3389/fvets.2020.542724)

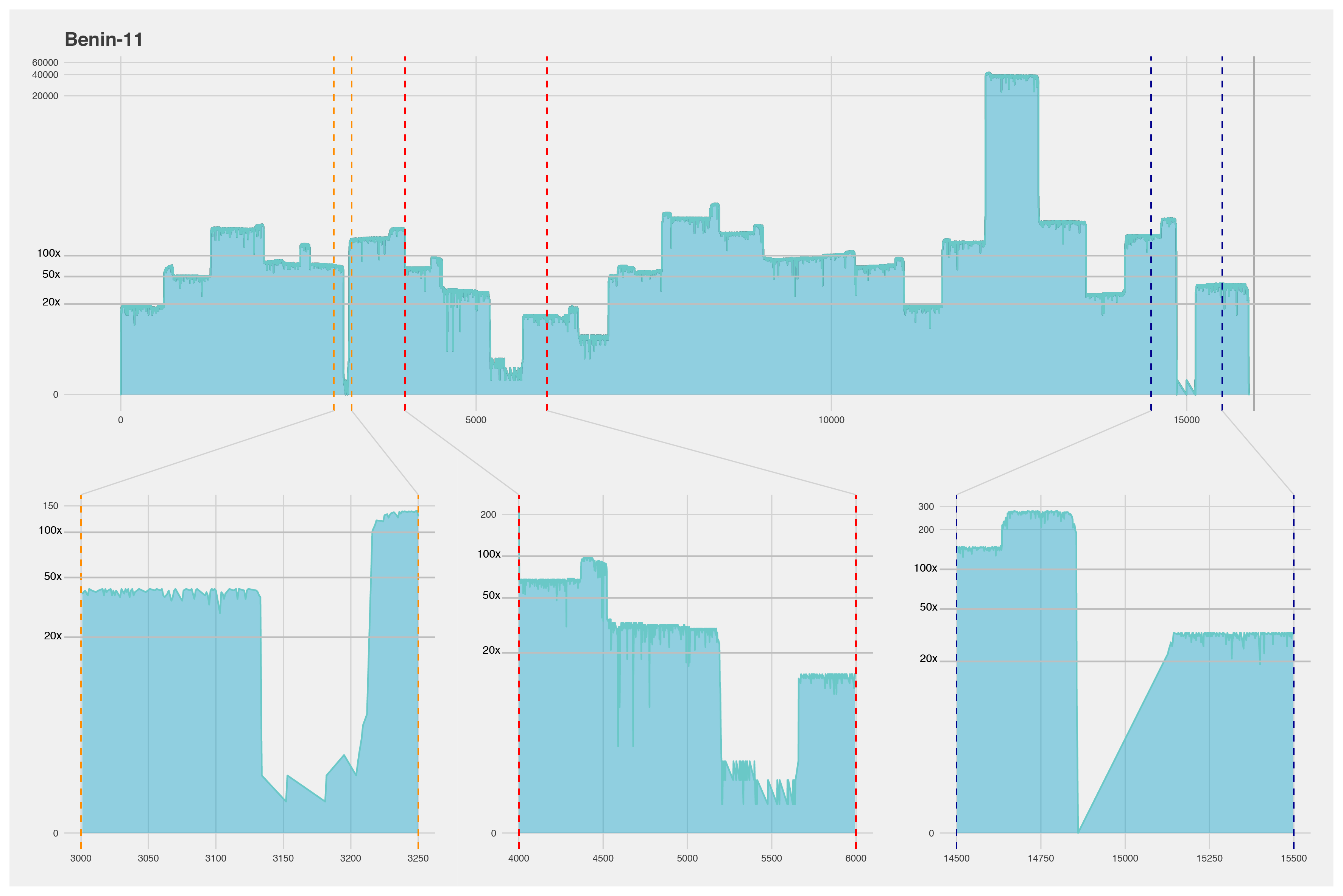

Supplement: Supplementary file 2 [file Image_1.TIF]

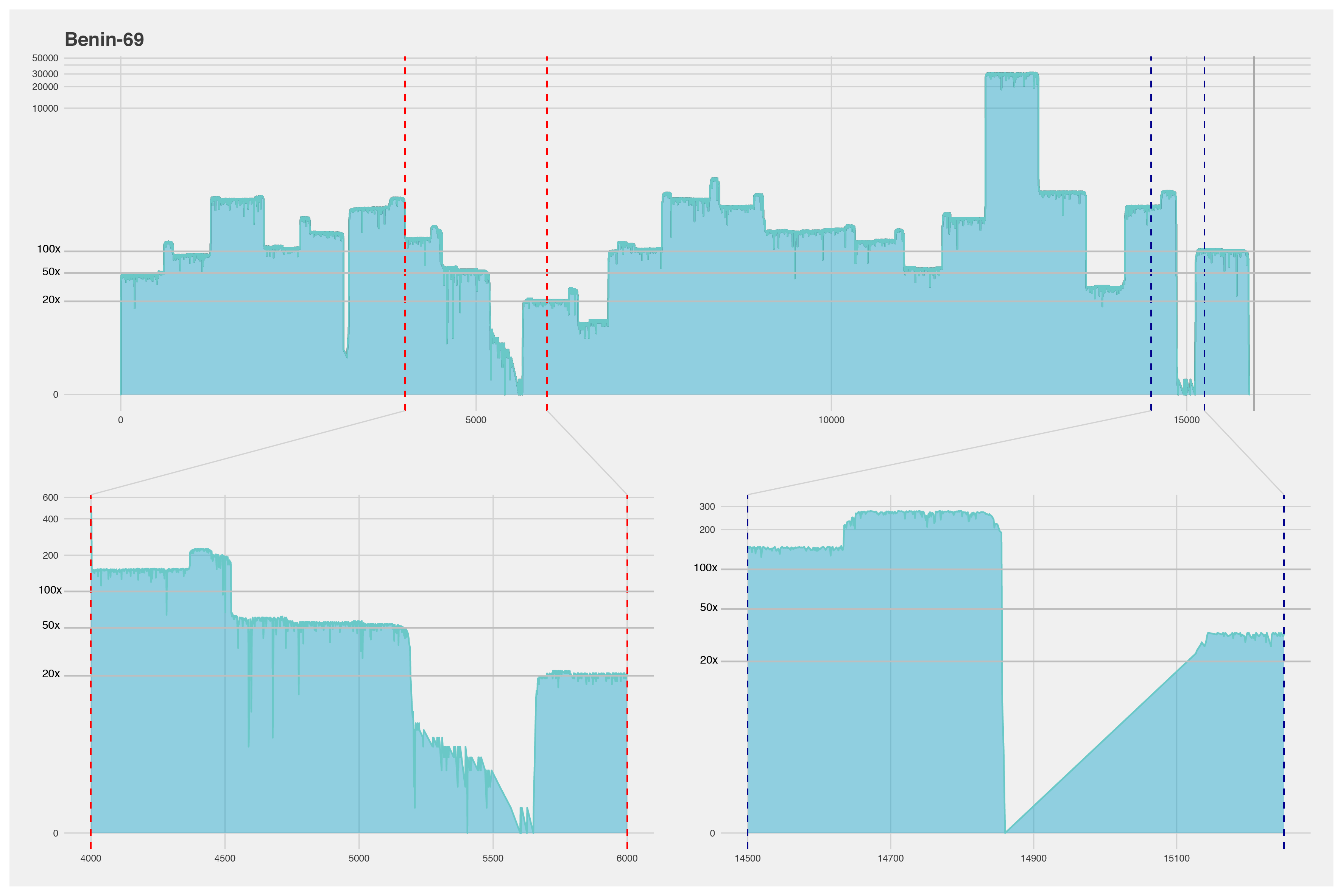

Supplement: Supplementary file 3 [file Image_2.TIF]

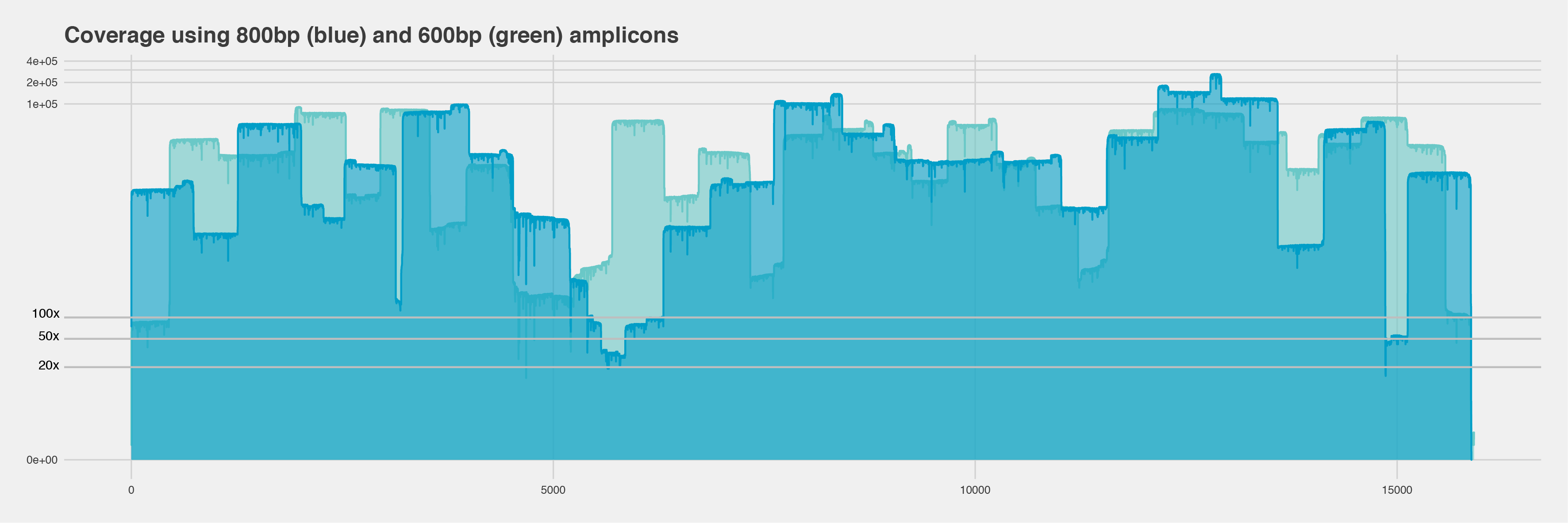

Supplement: Supplementary file 4 [file Image_3.TIF]

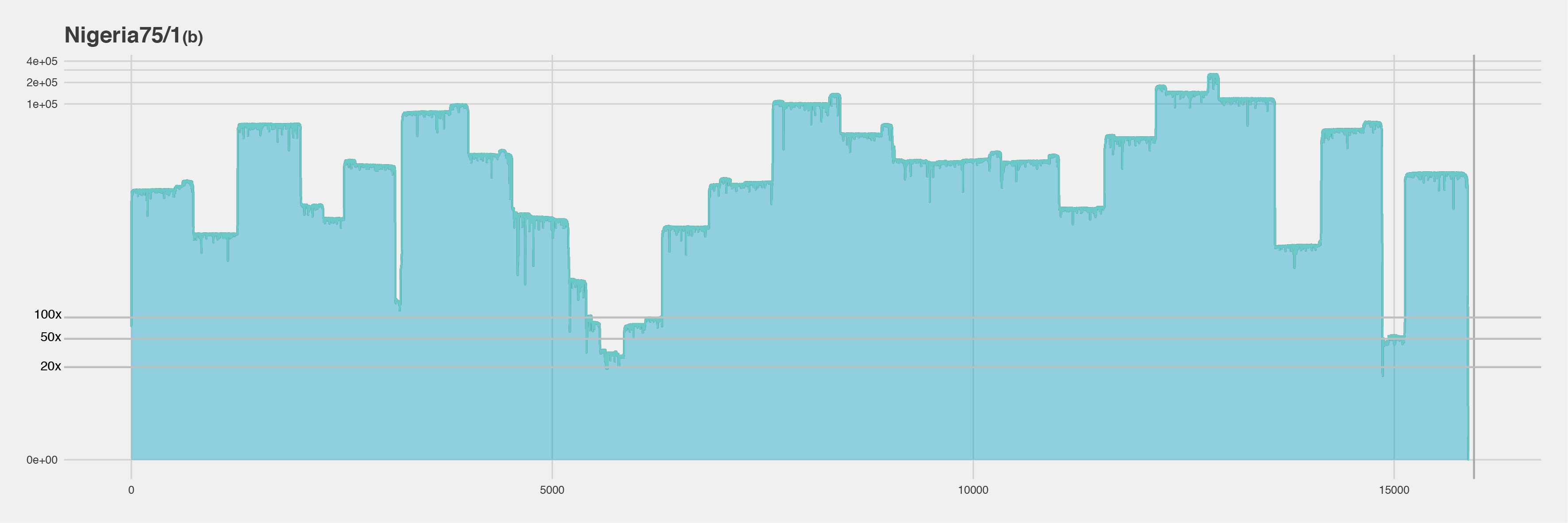

Supplement: Supplementary file 5 [file Image_4.TIF]

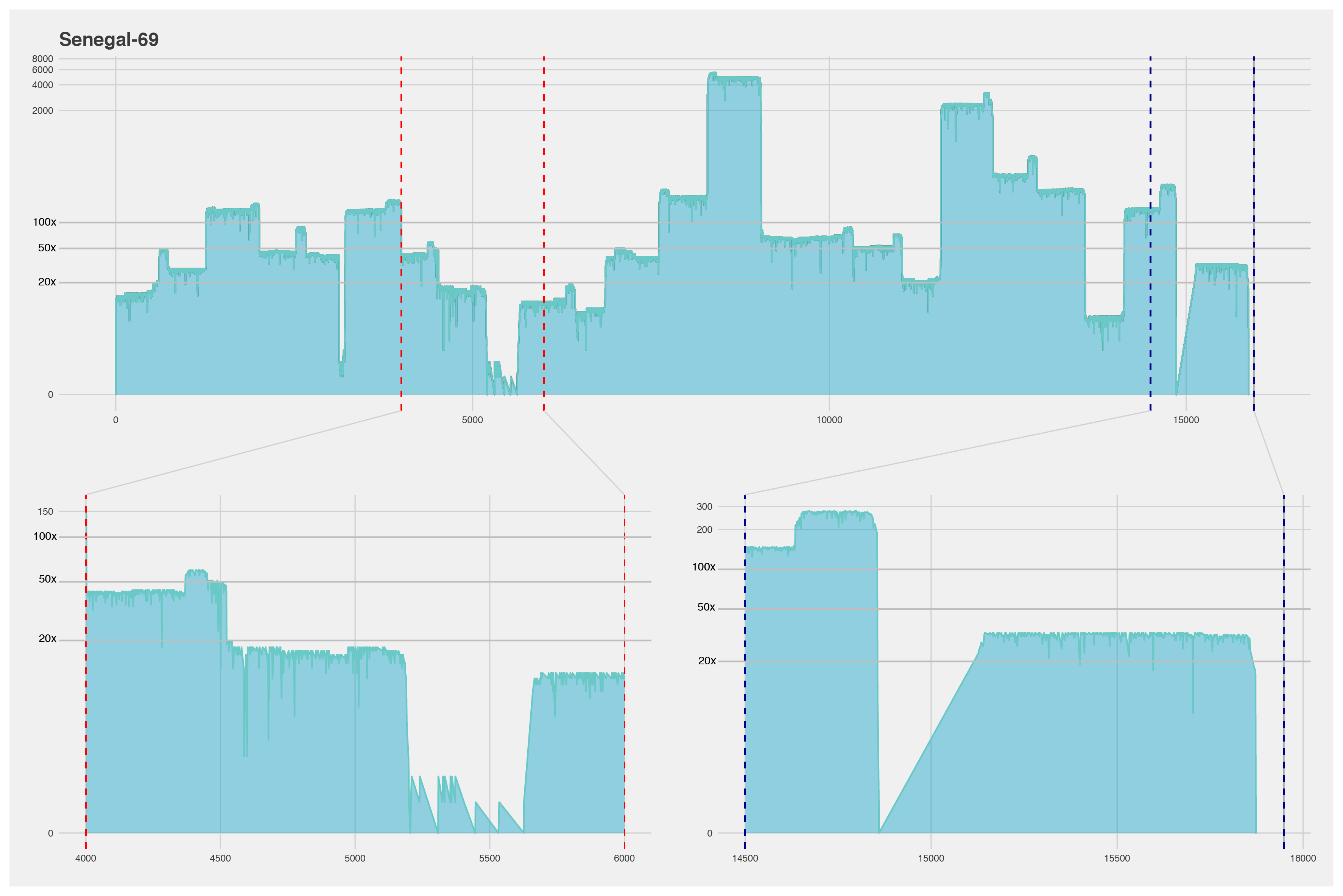

Supplement: Supplementary file 6 [file Image_5.TIF]

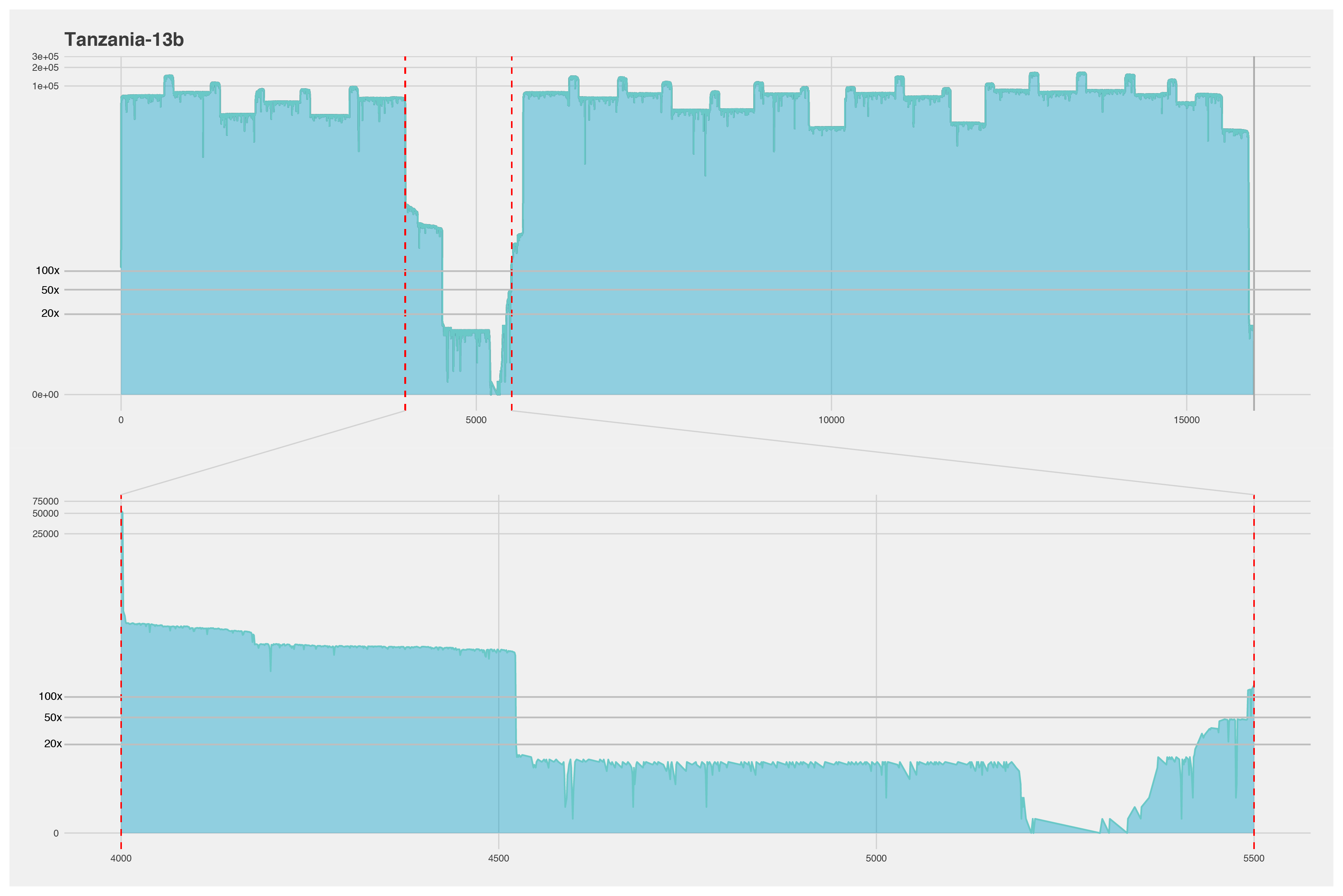

Supplement: Supplementary file 7 [file Image_6.TIF]

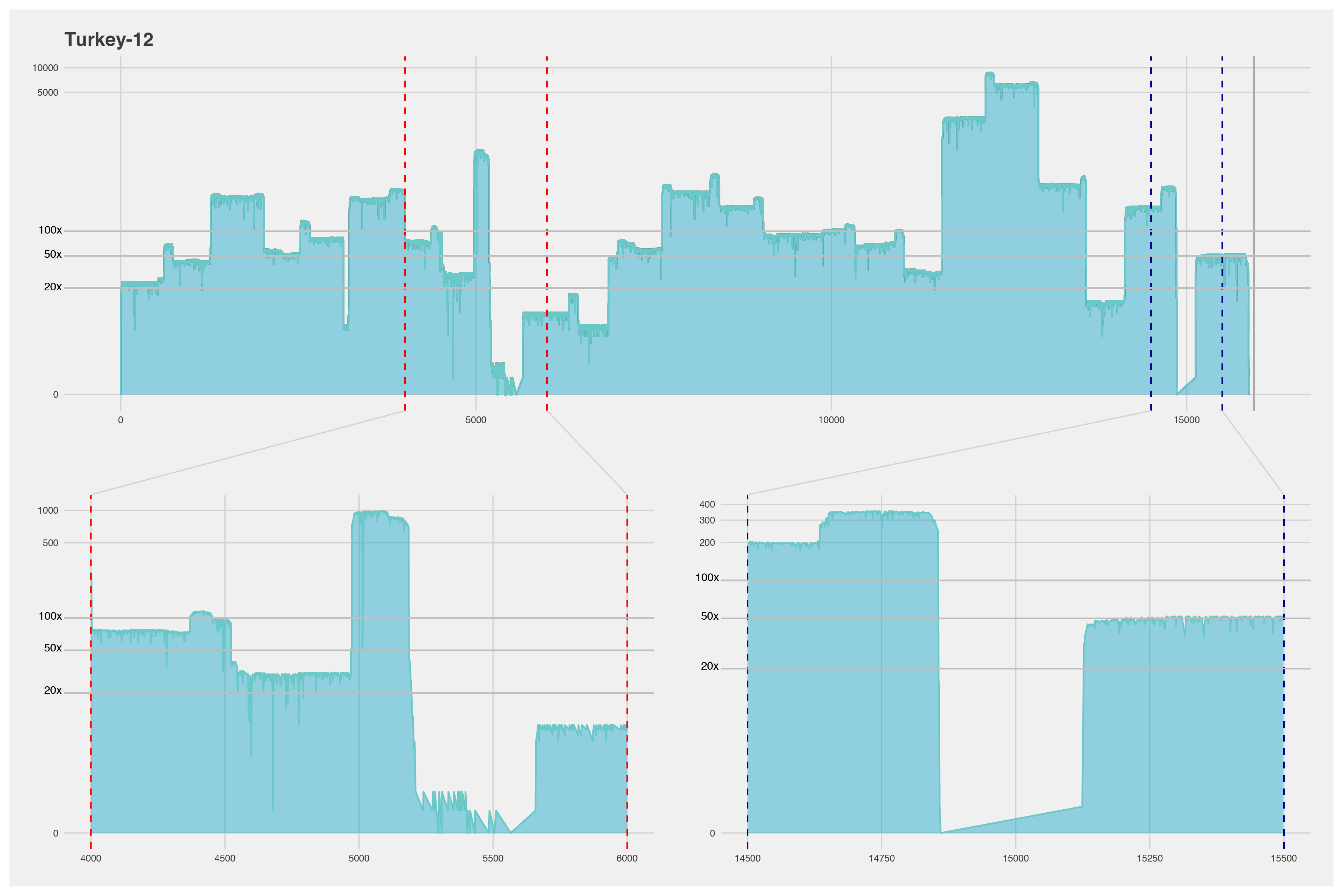

Supplement: Supplementary file 8 [file Image_7.TIF]

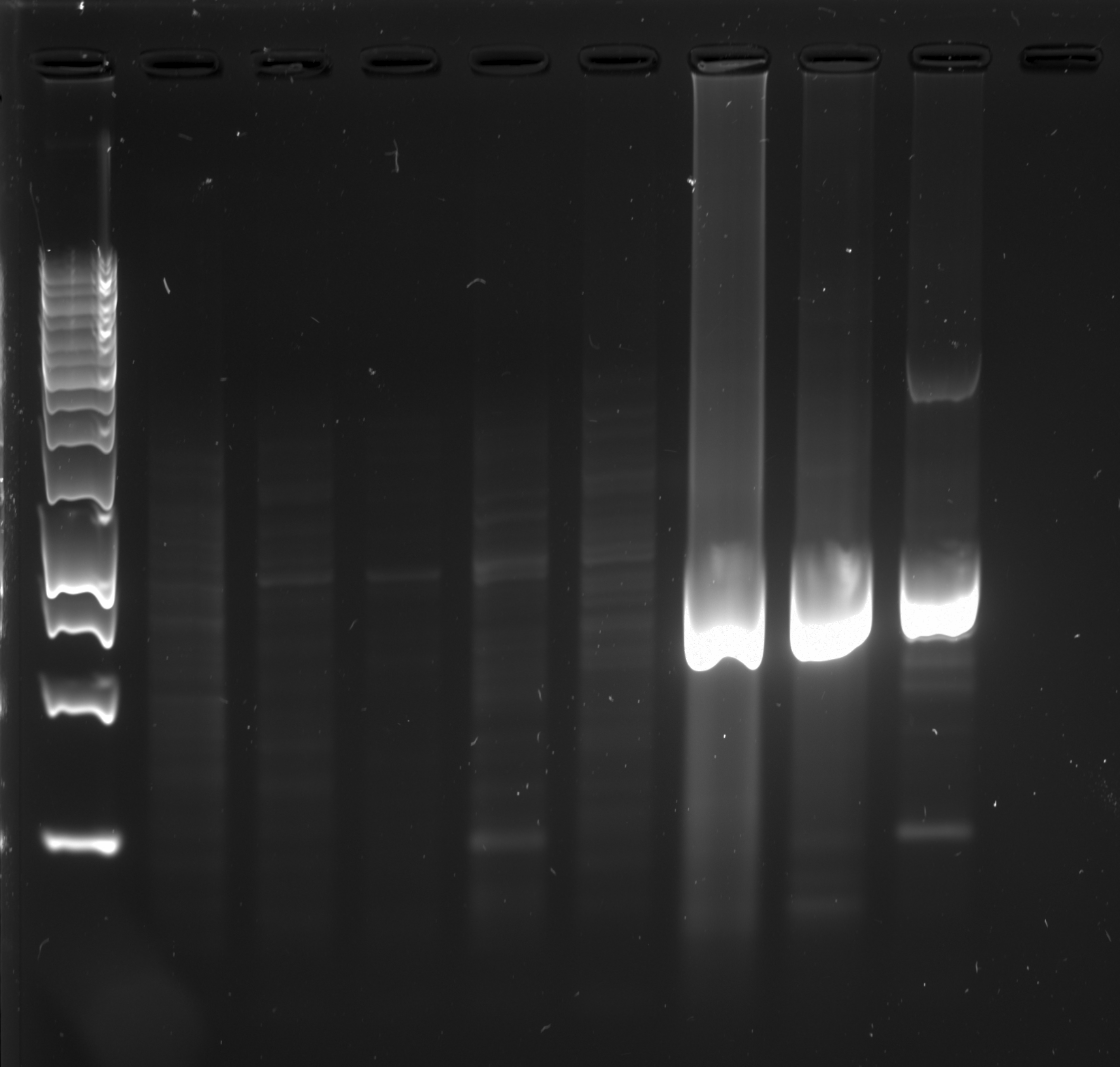

Supplement: Supplementary file 9 [file Image_8.TIF]
